# The relationship between IL-6 levels and the angiographic severity of coronary artery disease following percutaneous coronary intervention in acute coronary syndrome patients

**DOI:** 10.1186/s12872-021-02406-7

**Published:** 2021-12-03

**Authors:** Yang Ling, Hairong Weng, Shengxing Tang

**Affiliations:** 1 Department of Cardiology, Yijishan Hospital Affiliated To Wannan Medical College, 2# West Zhe Shan Road, Wuhu, 241000 China; 2 Department of Emergency Intensive Care Unit, Yijishan Hospital Affiliated To Wannan Medical College, 2# West Zhe Shan Road, Wuhu, 241000 China

**Keywords:** IL-6, Acute coronary syndrome, SYNTAX score, SYNTAX score II

## Abstract

**Background:**

The present investigation was developed for the exploration of the association between IL-6 levels and acute coronary syndrome (ACS) findings upon angiographic evaluation.

**Methods:**

A retrospective review of 346 patients suffering from chest discomfort that underwent coronary angiography was performed. The SYNergy between Percutaneous Coronary Intervention with TAXus and cardiac surgery (SYNTAX) score (SS) and SS II were used to gauge ACS severity, with ACS patients being stratified into two groups based on an SS value of 22 and the median SS II value. Associations between IL-6 levels and SS or SS II values were assessed through Spearman's correlation analyses, and independent predictors of intermediate-high SS or high SS II were identified via a multivariate logistic regression approach. A receiver operating characteristic (ROC) curve was employed to explore of the predictive value of IL-6 levels.

**Results:**

IL-6 was positively correlated with both SS (r = 0.479, P < 0.001) and SS II (r = 0.305, P < 0.001). Moreover, IL-6 levels were independently predictive of intermediate-high SS and high SS II values. ROC curves further demonstrated that IL-6 was able to predict intermediate-high SS and high SS II, with area under the curve (AUC) values of 0.806 and 0.624, respectively.

**Conclusion:**

IL-6 levels are closely linked to the extent of coronary artery disease in ACS patients undergoing percutaneous coronary intervention. IL-6 levels may thus serve as a valuable and non-invasive biomarker of high-risk ACS patients.

**Supplementary Information:**

The online version contains supplementary material available at 10.1186/s12872-021-02406-7.

## Background

Acute coronary syndrome (ACS) is a condition that incorporates several forms of myocardial ischemia, including ST-segment elevation myocardial infarction (STEMI), non-ST-segment elevation myocardial infarction (NSTEMI) as well as unstable angina pectoris (UA), and it is the leading cause for global morbidity and mortality [1, 2]. While improvements in antithrombotic treatment and revascularization approaches have improved the prognosis of ACS patients, many patients nonetheless experience unsatisfactory adverse cardiovascular outcomes [3]. At present consensus criteria pertaining to understanding ACS pathogenesis are lacking, and defining the optimal treatment for this condition is thus an important global public health goal [4].

A growing body of evidence suggests that inflammatory activity plays a maladaptive role in the context of ACS pathophysiology, triggering initiation and progression of atherosclerosis, driving plaque destabilization and degradation, and responding to myocardial necrosis [5–7]. Consistently, many studies have reported an association between cardiovascular disease (CVD) and increased levels of biomarkers indicative of inflammation [6, 8, 9]. In particular, the pro-inflammatory cytokine interleukin-6 (IL-6), which is primarily produced by macrophages and T cells, has been noted as a key driver of plaque destabilization, atheroprogression, and the production of high-sensitivity C-reactive protein (hs-CRP), leading to the development and progression of clinical atherosclerosis [6, 10–12]. Links between higher IL-6 levels and an elevated risk of cardiovascular events among otherwise healthy individuals are well documented [13, 14], and IL-6 has also been proposed to be a predictor of coronary artery disease (CAD) severity and associated mortality among ACS patients [6, 15, 16]. Additionally, levels of IL-6 have been reported to be associated with plaque burden as defined by intracoronary imaging [17].

In previous studies, the SYNergy between Percutaneous Coronary Intervention with TAXus and cardiac surgery (SYNTAX) score (SS), which is frequently used to quantify CAD degree and severity, has been shown to predict prognostic outcomes in stable CAD and ACS patients [18–20]. In recent years, the SS II indicator has been expanded to take individual clinical characteristics into account, reportedly achieving higher accuracy rates in the prognostic assessment of ACS patients [21, 22].

The relationships between IL-6 levels and both SS and SS II values, however, are poorly documented. As such, this study was designed to explore the link between IL-6 levels and ACS severity as measured using the SS and SS II indicators at the time of admission.

## Methods

### Study population

For this study, patients suffering from chest pain that were admitted to the Division of cardiology and underwent coronary angiography (CAG) between January 2021 to August 2021 were enrolled. The criteria for diagnosing ACS (including STEMI, NSTEMI, and UA patients) were based on the standard recommended by the ESC guidelines [23]. Patients were excluded if they had already undergone percutaneous coronary intervention (PCI) or coronary artery bypass grafting surgery (CABG), or if they exhibited malignancies, autoimmune disease, severe hepatic or renal failure, or infectious or inflammatory disease. In total, 346 patients were included in the final study, with these patients being classified into an ACS group and a stable angina pectoris (SAP) group (individuals with diseased vessels exhibiting > 50% luminal narrowing). This study was consistent with the Declaration of Heksinki and was approved by the Institutional Review Board of Yijishan Hospital Affiliated of Wannan Medical College.

### Patient's characteristics

The hospital electronic database was used to document all patient demographic and clinical characteristics. Fasting blood samples were obtained from the peripheral veins of all patients before PCI to assess hematologic indices, hs-CRP levels, biochemical parameters, and IL-6 concentrations using standard approaches in our hospital’s clinical laboratory. IL-6 concentrations were measured using an enzyme-linked immunosorbent assay (Human IL-6 ELISA Kit, Fine Test, Wuhan, China). Transthoracic echocardiography was conducted prior to angiography. The Cockcroft-Gault equation was utilized to calculate the estimated glomerular filtration rate (eGFR) for each patient.

### Coronary angiographic analysis

All patients underwent CAG via the radial approach at admission. CAG was performed by two expert interventional cardiologists who were blinded to patient clinical information. SS calculations were based on the coronary artery with a ≥ 50% luminal narrowing in a vessel ≥ 1.5 mm and were performed using the SS calculator (www.syntaxscore.com, version 2.1). Furthermore, SS II values were established based on these SS values, the presence of left main coronary artery disease, peripheral arterial disease (PAD), chronic obstructive pulmonary disease (COPD), female sex, eGFR, and left ventricular ejection fraction (LVEF) [24].

### Statistical analysis

The Kolmogorov–Smirnov approach was employed to assess whether continuous data were normally distributed. Normally distributed quantitative data are given as mean ± standard deviation (SD), while outcomes that were non-normally distributed are expressed as medians with the interquartile range. These outcomes were compared via Student's *t*-tests or Mann–Whitney *U*-tests. Categorical variables are given as numbers (percentages) and were compared using chi-squared tests or Fisher’s exact assessment. Parameters significant (P < 0.1) in initial univariate analyses were incorporated into a multivariate logistic regression analysis designed to identify independent predictors of intermediate-high SS and high SS II values. Receiver operating characteristic (ROC) curves were utilized to demonstrate the ability of IL-6 levels to predict these two outcomes. SPSS 23.0 was used for statistical analyses, and P < 0.05 was considered as statistically significant difference.

## Results

### Study participants

The demographic and clinical characteristics of ACS (n = 201) and SAP (n = 145) group patients were initially evaluated (Table 1). Overall, patients in the ACS group were mostly male and exhibited higher creatinine and fibrinogen levels (P < 0.05) relative to those of patients in the SAP group. Moreover, these ACS patients exhibited significant increases in white blood cell (WBC), neutrophil (NEUT), and platelet counts as well as neutrophil to the lymphocyte ratio (NLR) values compared to SAP patients (P < 0.05). Furthermore, IL-6 and hs-CRP concentrations were significantly greater in ACS group patients as compared to those in SAP group patients (P < 0.01), while apolipoprotein A1 (apoA1), high-density lipoprotein cholesterol (HDL-c), albumin, and LVEF were all reduced in these ACS patients (P < 0.05). No differences in age, body mass index (BMI), or other characteristics were observed when comparing these two groups. Additionally, no significant relationship between IL-6 levels and SS values in SAP patients were observed via Spearman’s correlation analyses (r = 0.128, P > 0.05; Additional file 1: Figure S1A).

**Table 1:** Demographic, clinical and biochemical characteristics between ACS and SAP group

Variables	ACS (n = 201)	SAP (n = 145)	P value
Age, years	65 (57–71)	65 (57–72)	.323
Male gender, n (%)	136 (67.7%)	83 (57.2%)	.047
Hypertension, n (%)	127 (63.2%)	95 (65.5%)	.655
Diabetes, n (%)	49 (24.4%)	27 (18.6%)	.202
Smoking, n (%)	30 (14.9%)	14 (9.7%)	.147
BMI, kg/m^2^	24.8 ± 3.3	24.7 ± 3.6	.916
WBC, 10^9^/L	6.7 (5.4–8.1)	5.9 (4.8–6.9)	< .001
NEUT, 10^9^/L	4.1 (3.4–5.6)	3.5 (2.7–4.4)	< .001
LYM, 10^9^/L	1.7 (1.3–2.1)	1.6 (1.3–2.0)	.293
NLR	2.4 (1.8–3.4)	2.1 (1.7–2.6)	.001
Platelet, 10^9^/L	175 (142–217)	166 (128–203)	.041
RDW	12.8 (12.5–13.4)	13.0 (12.6–13.3)	.470
Hemoglobin, g/L	129.5 ± 16.4	129.2 ± 15.7	.896
IL-6, pg/ml	7.5 (4.8–10.9)	3.8 (2.4–5.3)	< .001
hs-CRP, mg/l	6.0 (1.7–11.2)	1.2 (0.5–2.8)	< .001
Glucose, mmol/L	5.7 ± 2.3	5.4 ± 1.6	.202
TC, mmol/L	3.9 ± 1.0	3.9 ± 0.9	.858
Triglyceride, mmol/L	1.4 (1.0–1.9)	1.3 (0.9–1.8)	.280
HDL-c, mmol/L	1.2 (1.0–1.4)	1.3 (1.1–1.5)	.001
LDL-c, mmol/L	2.1 (1.6–2.8)	2.1 (1.5–2.7)	.706
apoB, g/L	0.8 (0.6–1.0)	0.8 (0.6–0.9)	.317
apoA1, g/L	1.1 (0.9–1.2)	1.2 (1.0–1.3)	< .001
Lp(a), mg/L	227.2 (102.5–469.4)	210.7 (90.5–386.5)	.208
Creatinine, umol/L	69.0 (57.7–85.7)	64.6 (53.7–76.0)	.012
eGFR, ml/min	86.2 (67.6–112.6)	91.4 (66.7–112.9)	.454
Albumin, g/L	39.0 ± 3.4	39.8 ± 3.5	.034
Fibrinogen, g/L	3.0 (2.6–3.9)	2.9 (2.4–3.2)	.001
D-Dimer, ug/ml	0.3 (0.2–0.4)	0.3 (0.2–0.5)	.066
LVEF, %	62.0 (58.0–65.0)	64.0 (60.0–66.0)	.001

ACS, acute coronary syndrome; SAP, stable angina pectoris; BMI, Body Mass Index; WBC, white blood cell; NEUT, neutrophil; LYM, lymphocyte; NLR, the neutrophil to the lymphocyte ratio; RDW, red cell distribution width; IL-6, interleukin 6; hs-CRP, high-sensitivity C-reactive protein; TC, total cholesterol; HDL-c, high-density lipoprotein cholesterol; LDL-c, low-density lipoprotein cholesterol; apoB, apolipoprotein B; apoA1, apolipoprotein A1; apoB/apoA1, the apoB to the apoA1 ratio; Lp(a), lipoprotein a; eGFR, estimated glomerular filtration rate; LVEF, left ventricular ejection fraction

### The relationship between IL-6 levels and an intermediate-high SYNTAX score

**Fig. 1:**
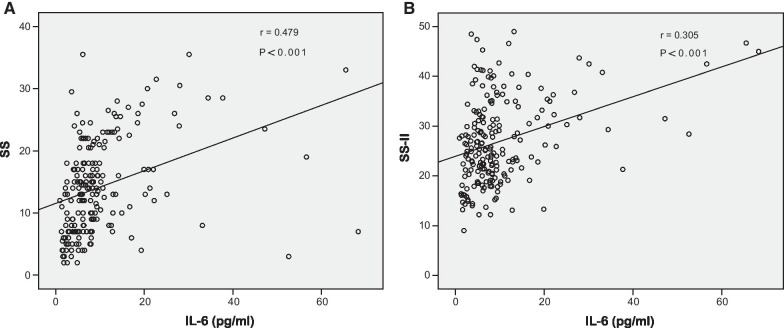
**A** Correlation between IL-6 levels and SS in ACS patients; **B** Correlation between IL-6 levels and SS II in ACS patients. IL-6, interleukin 6; SS, SYNergy between Percutaneous Coronary Intervention with TAXus and cardiac surgery (SYNTAX) score; SS II, SYNTAX score II; r, correlation coefficient

ACS patients were separated based on SS values cited in prior studies [25], with one group of patients with low SS values (SS ≤ 22, n = 168) and one group with intermediate-high SS values (SS > 22, n = 33). The demographic, clinical, biochemical, and angiographic parameters of ACS patients in these two groups are compiled in Table 2. IL-6 (P < 0.001) and hs-CRP (P < 0.001) levels in the intermediate-high SS group were significantly elevated in comparison to those in the low SS group, and the SS II and residual SS (rSS) values for cases in the intermediate-high SS group were also greater compared to those in the low SS group (P < 0.05). Spearman's correlation analyses revealed IL-6 levels and SS values to be significantly positively associated with one another (r = 0.479, P < 0.001; Fig. 1A). Additionally, IL-6 levels were not correlated with post-PCI troponin values (r = 0.107, P > 0.05; Additional file 1: Figure S1B).

**Table 2:** Demographic, clinical, biochemical and angiographic characteristics in low and intermediate-high SYNTAX score (SS) group

Variables	SS ≤ 22 (n = 168)	SS > 22 (n = 33)	P value
Age, years	65 (57–71)	63 (58–75)	.304
Male gender, n (%)	114 (67.9%)	22 (66.7%)	.894
Hypertension, n (%)	107 (63.7%)	20 (60.6%)	.737
Diabetes, n (%)	40 (23.8%)	9 (27.3%)	.672
Smoking, n (%)	25 (14.9%)	5 (15.2%)	.968
BMI, kg/m^2^	24.9 ± 3.5	24.2 ± 2.7	.312
WBC, 10^9^/L	6.6 (5.3–8.0)	7.0 (5.6–8.8)	.210
NEUT, 10^9^/L	4.1 (3.3–5.4)	4.5 (3.6–5.9)	.231
LYM, 10^9^/L	1.7 (1.3–2.1)	1.9 (1.3–2.2)	.833
NLR	2.4 (1.8–3.4)	2.6 (2.0–3.6)	.277
Platelet, 10^9^/L	174 (141–212)	185 (145–225)	.275
RDW	12.9 (12.5–13.3)	12.8 (12.5–13.4)	.741
Hemoglobin, g/L	129.8 ± 14.6	128.1 ± 23.7	.597
IL-6, pg/ml	6.5 (4.3–9.2)	14.0 (11.1–24.8)	< .001
hs-CRP, mg/l	4.5 (1.3–9.0)	11.8 (8.7–20.0)	< .001
Glucose, mmol/L	5.7 ± 2.5	5.6 ± 1.4	.830
TC, mmol/L	3.9 ± 1.0	4.0 ± 1.0	.578
Triglyceride, mmol/L	1.4 (1.0–2.0)	1.6 (1.2–1.8)	.409
HDL-c, mmol/L	1.2 (1.1–1.4)	1.1 (1.0–1.3)	.187
LDL-c, mmol/L	2.2 ± 0.8	2.4 ± 0.9	.176
apoB, g/L	0.8 (0.6–0.9)	0.9 (0.6–1.1)	.138
apoA1, g/L	1.0 ± 0.2	1.1 ± 0.2	.571
Creatinine, umol/L	69.4 (57.7–85.2)	68.5 (57.2–87.6)	.984
eGFR, ml/min	86.3 (67.4–113.1)	86.1 (66.8–104.2)	.570
Albumin, g/L	39.4 (37.5–41.5)	38.6 (35.9–40.6)	.091
Fibrinogen, g/L	3.0 (2.6–3.8)	3.4 (2.8–4.0)	.129
D-Dimer, ug/ml	0.3 (0.2–0.5)	0.4 (0.2–0.6)	.400
LVEF, %	62.0 (58.0–65.0)	62.0 (53.0–65.0)	.744
SS	12.0 (7.0–15.8)	26.0 (23.5–28.5)	< .001
SS II	24.5 (19.9–31.3)	30.5 (24.5–37.5)	.001
rSS	2.0 (0–5.0)	10.0 (4.0–16.8)	< .001

BMI, Body Mass Index; WBC, white blood cell; NEUT, neutrophil; LYM, lymphocyte; NLR, the neutrophil to the lymphocyte ratio; RDW, red cell distribution width; IL-6, interleukin 6; hs-CRP, high-sensitivity C-reactive protein; TC, total cholesterol; HDL-c, high-density lipoprotein cholesterol; LDL-c, low-density lipoprotein cholesterol; apoB, apolipoprotein B; apoA1, apolipoprotein A1; apoB/apoA1, the apoB to the apoA1 ratio; eGFR, estimated glomerular filtration rate; LVEF, left ventricular ejection fraction; SYNTAX, SYNergy between Percutaneous Coronary Intervention with TAXus and cardiac surgery; SS, SYNTAX score; SS II, SYNTAX score II; rSS, residual SYNTAX score

### Identification of independent predictors of intermediate-high SYNTAX score

**Fig. 2:**
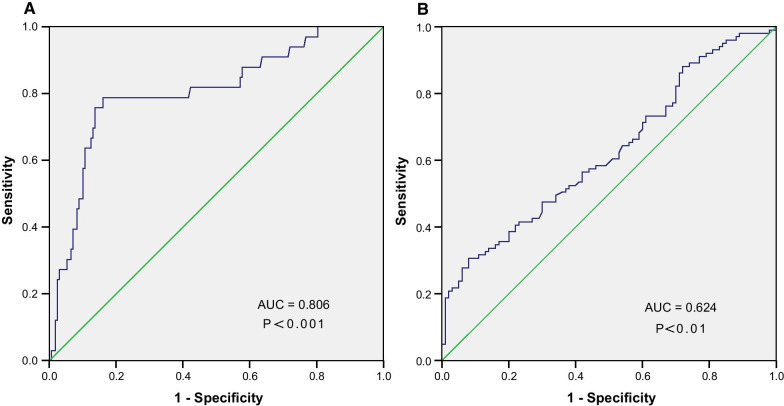
**A** The receiver operating characteristic (ROC) cure of IL-6 in predicting SS > 22 in ACS patients; **B** ROC curve of IL-6 in predicting SS II > 25.4 in ACS patients. AUC, area under the curve; SS, SYNergy between Percutaneous Coronary Intervention with TAXus and cardiac surgery (SYNTAX) score; SS II, SYNTAX score II

Multivariable logistic regression analyses were next conducted to explore independent predictors of intermediate-high SS by analyzing all variables that exhibited significant predictive value (P < 0.1) in univariate assessment, including, IL-6, hs-CRP, and albumin. The outcomes of this analysis revealed that serum IL-6 levels (odds ratio [OR] = 1.081, 95% confidence interval [CI]: 1.036–1.128, P < 0.001) were independent predictors of intermediate-high SS (Table 4). Consistently, an ROC curve analysis for IL-6 yielded an AUC of 0.806 (95% CI: 0.718–0.895, P < 0.001; Fig. 2A) when used to predict intermediate-high SS values, with an optimal predictive cut-off value of 10.6 pg/ml, yielding respective sensitivity and specificity values of 78.79% and 83.93%.

### Factors related to high SS II

Median SS II values were next used to stratify all ACS patients into the low SS II (SS II ≤ 25.4; n = 100) and high SS II (SS II > 25.4; n = 101) groups. Those cases in the high SS II group were, on average, older, had a higher IL-6 value, and exhibited lower hemoglobin levels, eGFR, albumin, and BMI values as compared to individuals in the low SS II group (P < 0.01; Table 3). Additionally, no differences in rSS, history of hypertension or diabetes, or other characteristics were observed when comparing these two groups. IL-6 levels were also positively associated with SS II (r = 0.305, P < 0.001; Fig. 1B). After univariate analyses of associated parameters, several variables, including IL-6, hs-CRP, hemoglobin, and NLR, were then incorporated into a multivariable logistic regression analysis, which revealed that IL-6 levels (OR = 1.082, 95% CI: 1.025–1.143, P < 0.01) and hemoglobin levels (OR = 0.948, 95% CI: 0.924–0.972, P < 0.001) were independent predictors of high SS II values (Table 4). Consistently, ROC curves demonstrated the value of IL-6 as a predictor of high SS II (AUC = 0.624, P < 0.01; Fig. 2B). An IL-6 > concentration greater than 12.9 pg/ml was predictive of high SS II, with sensitivity and specificity of 30.69% and 92.00%, respectively.

**Table 3:** Demographic, clinical, biochemical and angiographic characteristics in low and high SYNTAX score II group

Variables	SS II ≤ 25.4 (n = 100)	SS II > 25.4 (n = 101)	P value
Age, years	57 (50–65)	70 (65–75)	< .001
Male gender, n (%)	85 (85.0%)	51 (50.5%)	< .001
Hypertension, n (%)	57 (57.0%)	70 (69.3%)	.070
Diabetes, n (%)	28 (23.0%)	21 (20.8%)	.234
Smoking, n (%)	23 (25.0%)	7 (6.9%)	.001
COPD, n (%)	2 (2.0%)	2 (2.0%)	1.0
PAD, n (%)	5 (5.0%)	8 (7.9%)	.400
BMI, kg/m^2^	25.7 ± 3.6	23.9 ± 2.9	< .001
WBC, 10^9^/L	6.7 (5.5–8.4)	6.5 (5.3–8.0)	.383
NEUT, 10^9^/L	4.1 (3.4–5.4)	4.1 (3.4–5.7)	.964
LYM, 10^9^/L	1.9 (1.4–2.2)	1.6 (1.2–2.0)	.007
NLR	2.3 (1.7–3.3)	2.6 (2.0–3.5)	.069
Platelet, 10^9^/L	188 (148–222)	162 (140–210)	.061
RDW	12.8 (12.5–13.3)	13.0 (12.6–13.5)	.053
Hemoglobin, g/L	135.3 ± 15.4	123.7 ± 15.4	< .001
IL-6, pg/ml	6.6 (3.8–9.2)	8.2 (5.2–14.4)	.002
hs-CRP, mg/l	5.7 (1.2–10.1)	6.2 (2.4–14.7)	.087
Glucose, mmol/L	5.8 ± 2.8	5.5 ± 1.8	.336
TC, mmol/L	4.0 ± 1.0	3.9 ± 1.0	.552
Triglyceride, mmol/L	1.5 (1.0–2.0)	1.3 (1.0–1.9)	.439
HDL-c, mmol/L	1.2 (1.0–1.4)	1.2 (1.1–1.4)	.213
LDL-c, mmol/L	2.2 ± 0.7	2.2 ± 0.8	.715
apoB, g/L	0.8 (0.6–1.0)	0.8 (0.6–0.9)	.662
apoA1, g/L	1.0 ± 0.2	1.1 ± 0.2	.321
Creatinine, umol/L	68.4 (59.2–79.6)	72.0 (55.4–92.7)	.237
eGFR, ml/min	103.9 (86.1–126.9)	69.9 (53.9–88.8)	< .001
Albumin, g/L	39.9 (38.4–42.2)	38.1 (36.1–40.5)	< .001
Fibrinogen, g/L	3.0 (2.5–3.6)	3.2 (2.7–4.0)	.041
D-Dimer, ug/ml	0.3 (0.2–0.4)	0.4 (0.2–0.7)	.001
LVEF, %	63.0 (59.0–65.0)	62.0 (50.0–65.0)	.050
SS	11.0 (7.0–16.0)	15.0 (9.0–22.0)	< .001
SS II	20.8 (17.8–22.9)	32.3 (28.6–38.1)	< .001
rSS	2.0 (0–5.4)	4.0 (0–9.0)	.161

BMI, Body Mass Index; WBC, white blood cell; NEUT, neutrophil; LYM, lymphocyte; NLR, the neutrophil to the lymphocyte ratio; RDW, red cell distribution width; IL-6, interleukin 6; hs-CRP, high-sensitivity C-reactive protein; TC, total cholesterol; HDL-c, high-density lipoprotein cholesterol; LDL-c, low-density lipoprotein cholesterol; apoB, apolipoprotein B; apoA1, apolipoprotein A1; apoB/apoA1, the apoB to the apoA1 ratio; eGFR, estimated glomerular filtration rate; LVEF, left ventricular ejection fraction; SYNTAX, SYNergy between Percutaneous Coronary Intervention with TAXus and cardiac surgery; SS, SYNTAX score; SS II, SYNTAX score II

**Table 4:** Independent predictors of intermediate–high SYNTAX score (> 22) and high SYNTAX score II (> 25.4)

	Univariate analysis				Multivariable analysis			
	***P***	OR	95% CI		***P***	OR	95% ci	
Independent predictors of intermediate–high SYNTAX score								
IL-6	< .001	1.085	1.043	1.130	< .001	1.081	1.036	1.128
hs-CRP	< .001	1.052	1.023	1.082	.056	1.032	0.999	1.066
Albumin	.055	0.912	0.831	1.002	.154	0.921	0.822	1.031
Independent predictors of high SYNTAX score II								
hs-CRP	.0063	1.025	0.999	1.052	.670	0.993	0.962	1.026
Hemoglobin	< .001	0.948	0.927	0.970	< .001	0.948	0.924	0.972
IL-6	.001	1.085	1.034	1.138	.005	1.082	1.025	1.143
NLR	.087	1.108	0.985	1.247	.480	1.050	0.917	1.203

IL-6, interleukin 6; hs-CRP, high-sensitivity C-reactive protein; NLR, the neutrophil to the lymphocyte ratio; OR, odds ratio; SYNTAX, SYNergy between Percutaneous Coronary Intervention with TAXus and cardiac surgery

## Discussion

ACS remains the most prominent threat to global public health, despite advances in revascularization techniques and antithrombotic therapy [1, 3]. It is thus essential that approaches to reliably predicting ACS severity be developed in order to guide the prevention, diagnosis and treatment of this debilitating disease. Herein, we found that IL-6 levels were positively correlated with ACS severity as measured by SS and SS II, with IL-6 levels additionally offering value as an independent predictor of intermediate-high SS and high SS II values.

A growing body of evidence suggests that inflammation is a key driver of ACS onset, progression, and patient prognosis [5–7]. Likewise, cytokines, regarded as the “messengers” of inflammatory response, have been implicated in the pathogenesis of atherosclerosis and CAD [6, 8, 26]. Of note, IL-6, which is primarily derived from mononuclear cells, can contribute to CAD initiation and progression through several mechanisms. Not only does IL-6 mainly initiate the production of hepatic CRP, resulting in increased blood viscosity and platelets numbers; it also accelerates the deposition of fibrinogen [16]. IL-6 can further stimulate macrophages to phagocytose lipids, thereby driving foam cell formation [27]. There is also evidence to suggest that IL-6 is capable of activating the hypothalamic–pituitary–adrenal axis and accelerating insulin resistance [28].

In prior studies, higher IL-6 levels in healthy males were associated with future myocardial infarction incidence [13]. Additionally, Ikeda et al. demonstrated that ACS patients exhibit substantially higher levels of circulating IL-6 as compared to stable angina patients [29]. Moreover, CAD patients exhibit higher serum IL-6 concentrations as compared to controls [30]. One previous study reported that no statistically significant differences in IL-6 levels were observed in the blood of ACS patients taken from the coronary sinus in comparison to blood taken from a peripheral vein, supporting the concept of a systemic rather than a local vascular inflammation contributing to the development of atherosclerosis [31]. Herein, we similarly observed higher serum IL-6 concentrations in ACS patients relative to those in the SAP group.

The SS is a valuable tool that can guide appropriate revascularization planning by aiding in the detection of high-risk ACS cases, and it is also linked to the complexity of atherosclerotic lesions. Furthermore, SS values can effectively predict adverse cardiovascular event risk [18–20]. Additionally, several studies have demonstrated that a correlation exists between IL-6 levels and the severity of coronary stenoses and mortality [6, 16]. Indeed, there have also been prior reports of a positive association between IL-6 levels and the severity of CAD as assessed based on the Gensini score, which is one of the most common scoring systems for quantifying CAD severity [26, 32]. These prior results suggested a likely correlation between serum IL-6 levels and ACS severity as measured using SS and SS II values. Consistently, we found that IL-6 concentrations were independently predictive of intermediate-high SS values, with which they were positively correlated. Given that a range of clinicopathological variables can influence patient prognosis, the SS II scoring system was developed as a more reliable predictor of cardiovascular events among ACS patients by taking these variables into consideration [21, 22]. We detected a positive relationship between IL-6 levels and SS II values. Moreover, we identified hemoglobin concentrations and IL-6 levels to be independent predictors of high SS II. This relationship between hemoglobin and SS II values was in accordance with a previous study [24]. Other reports have also found that factors, including older age, decreased LVEF, and eGFR, can increase SS II values [33–35]. Patients with anemia more frequently present with advanced age, higher creatinine levels, and LVEF dysfunction [36, 37]. Therefore, the association between decreased hemoglobin and a high SS may be attributable to older age, reduced eGFR, and lower LVEF in these patients.

## Limitations

There are certain limitations to the present study. For one, a high degree of variability with respect to the IL-6 levels of ACS patients was observed, suggesting that measurements taken at a single time point may be insufficient to reliably capture the extent of potential inflammatory activity. Besides that, we examined the association between the IL-6 and SS or SS II, however, it is difficult to make causal inferences due to the nature of the cross-sectional design. A mendelian randomization study may be further designed to explore this association. Additionally, this was a retrospective single-center analysis without the potential to avoid selection bias. Several confounding factors might have affected the results even after the adjusted analysis. Furthermore, data regarding SS values < 30 and IL-6 levels may be skewed, thus weakening the observed correlations. Lastly, this was a retrospective analysis with relatively few samples, underscoring the need for future large-scale prospective analyses designed to validate and expand upon these results.

## Conclusions

In summary, this study confirmed the association between the levels of IL-6 and angiographic complexity in ACS patients. Overall, these findings suggest that IL-6 may be a biomarker that can be used to gauge CAD severity.

## Supplementary Information

**Additional file 1. Figure S1**. (A) Relationship between IL-6 levels and SS in SAP patients; (B) Relationship between IL-6 levels and Post-TnI in SAP patients. IL-6, interleukin 6; SS, SYNergy between Percutaneous Coronary Intervention with TAXus and cardiac surgery (SYNTAX) score; SAP, stable angina pectoris; r, correlation coefficient

## Data Availability

The raw data supporting the conclusions of this article will be made available by the corresponding authors, without undue reservation.

## Abbreviations

ACS: Acute coronary syndrome
SYNTAX: SYNergy between Percutaneous Coronary Intervention with TAXus and cardiac surgery
SS: SYNTAX score
ROC: Receiver operating characteristic
AUC: Area under the curve
STEMI: ST-segment elevation myocardial infarction
NSTEMI: Non-ST-segment elevation myocardial infarction
UA: Unstable angina pectoris
CVD: Cardiovascular disease
IL-6: Interleukin-6
hs-CRP: High-sensitivity C-reactive protein
CAD: Coronary artery disease
CAG: Coronary angiography
PCI: Percutaneous coronary intervention
CABG: Coronary artery bypass grafting surgery
SAP: Stable angina pectoris
eGFR: Estimated glomerular filtration rate
PAD: Peripheral arterial disease
COPD: Chronic obstructive pulmonary disease
LVEF: Left ventricular ejection fraction
SD: Standard deviation
WBC: White blood cell
NEUT: Neutrophil
NLR: The neutrophil to the lymphocyte ratio
apoA1: Apolipoprotein A1
HDL-c: High-density lipoprotein cholesterol
BMI: Body mass index
OR: Odds ratio
CI: Confidence interval
