# Supplementary figures and images for: The relationship between IL-6 levels and the angiographic severity of coronary artery disease following percutaneous coronary intervention in acute coronary syndrome patients
Source: BMC Cardiovasc Disord. 2021 Dec 3;21:578. doi: 10.1186/s12872-021-02406-7 (PMC8642871; doi:10.1186/s12872-021-02406-7)

**A**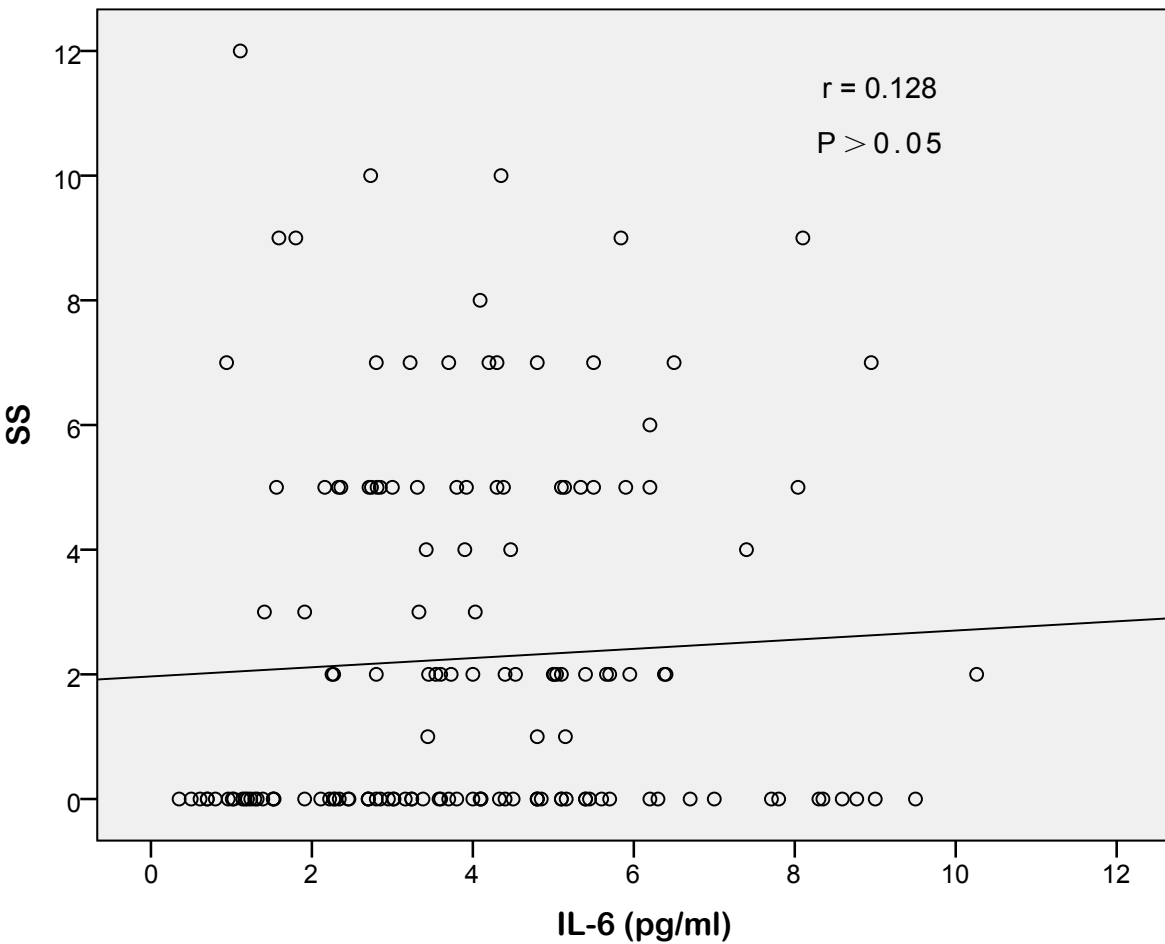**B**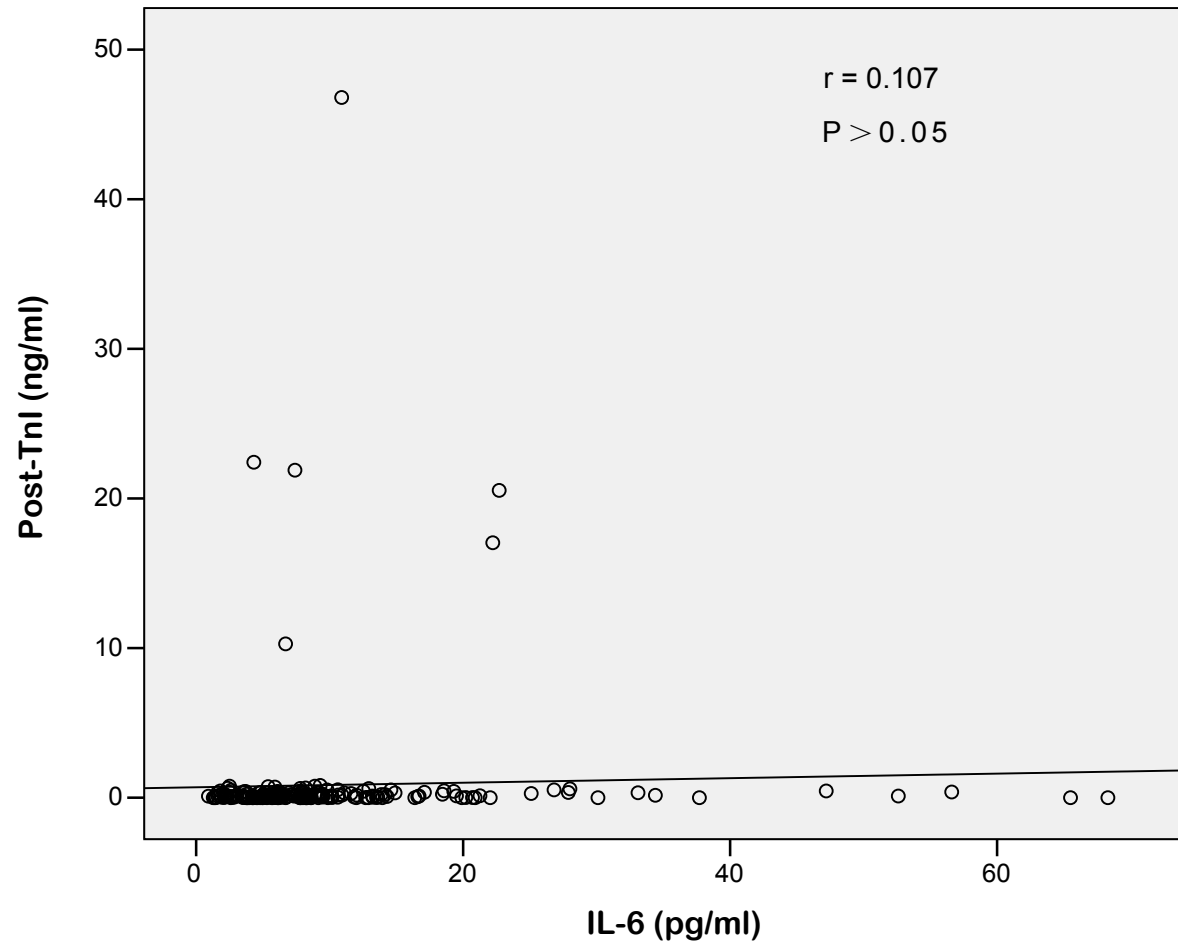

Supplement: Supplementary file 1 — Additional file 1. Figure S1. (A) Relationship between IL-6 levels and SS in SAP patients; (B) Relationship between IL-6 levels and Post-TnI in SAP patients. IL-6, interleukin 6; SS, SYNergy between Percutaneous Coronary Intervention with TAXus and cardiac surgery (SYNTAX) score; SAP, stable angina pectoris; r, correlation coefficient [file 12872_2021_2406_MOESM1_ESM.pdf]
